# Skin microcirculation and hypertension: is there a connection?

**DOI:** 10.1590/2175-8239-JBN-2024-0192en

**Published:** 2025-06-02

**Authors:** Jackeline Flores, Camilo Pena, Kenneth Nugent

**Affiliations:** 1 Texas Tech University Health Sciences Center, School of Medicine, Department of Internal Medicine, Lubbock, Texas, United States.

**Keywords:** Sodium, Capillary Resistance, Hypertension, Microvascular Rarefaction

## Abstract

**Introduction:**

Hypertension is an important risk factor for myocardial infarction, stroke, peripheral arterial disease, and chronic kidney disease. The World Health Organization has determined that approximately 1.28 billion adults worldwide have high blood pressure. Its definition has changed over the years, and in 2017 the American College of Cardiology and the American Heart Association have defined blood pressures ≥ 130/80 mmHg as hypertension.

**Methods:**

The PubMed database was searched using the MeSH terms sodium, capillary resistance, hypertension, and microvascular rarefaction to identify articles relevant to skin sodium levels, peripheral circulations and vascular rare fraction, and hypertension.

**Results:**

Experimental animal and human studies indicate that there are fewer capillaries and arterioles in patients with primary hypertension, and decreased density, also known as rarefaction, increases peripheral resistance. Microvascular density can be estimated noninvasively by methods such as intravital video microscopy in cutaneous regions or in nailfolds. The mechanisms of the reduction in density have been studied and could be used as a parameter when evaluating therapeutic options for patients. The skin provides a reservoir for sodium and can help moderate fluctuations in blood pressure associated with changes in sodium intake. In addition, the skin can modulate fluid volumes in the body through transepidermal water loss.

**Conclusions:**

This review discusses microvascular rarefaction, changes in microvasculature structure including the skin capillaries with hypertension, the association between sodium and skin physiology relevant to hypertension, transepidermal water loss, and the vascular effects associated with some antihypertensive medications that help explain their benefit in the prevention of cardiovascular events.

## Introduction

One in every two people older than 20 years in the United States has hypertension, and only 39.6% of hypertensive patients on medications has well-controlled blood pressure^1^. Multiple factors contribute to the development of and are associated with hypertension; these include the autonomic nervous system, the renin-angiotensin-aldosterone system, the vasopressin system, and environmental factors, such as salt intake and stress. Initial events likely involve the microcirculation; chronic increases in blood pressure develop secondary to changes in the microcirculation, including vasoconstriction, tissue hypoxia, and reduced arteriolar and capillary density. These events increase peripheral vascular resistance, which can cause tissue injury, starting with microvascular adaptations leading to organ damage^2^. The skin contains small arterioles and venules and non-osmotic sodium, and patients with hypertension have higher levels of skin sodium storage measured with sodium-23 magnetic resonance imaging (^23^Na-MRI)^3,4^. Consequently, studies on the skin microcirculation and its sodium levels can provide both insights and opportunities to study hypertension. Key findings on these mechanisms are summarized in Chart 1.

**Chart 1:** Key statements about the microcirculation and sodium content in the skin

1. The skin contains a complex network of arterioles, capillaries, and venules. Anatomic changes in the arterioles and their density in the skin and physiological responses to vasodilators and vasoconstrictors can affect peripheral vascular resistance and blood pressure.
2. Glycosaminoglycans (GAGs) in the dermis bind sodium and provide a non-osmotic storage site for sodium in the body.
3. Increased sodium levels in the skin can decrease blood vessel density and increase GAGs content, inflammatory cell influx, and lymph vessel density.
4. Microvascular rarefaction can occur secondary to functional changes in vessels with vasoconstriction and structural changes in vessels with chronic remodeling. These changes can often occur early in hypertension and increase blood pressure.
5. Transepidermal water loss can help modulate fluid volumes in the body and may work in a reciprocal manner with the kidney.
6. Antihypertensive medications, such as dihydropyridine calcium channel blockers, angiotensin-converting enzyme (ACE) inhibitors, and angiotensin II receptor blockers, can decrease large artery stiffness and reverse microvascular remodeling.
7. Current experimental methods can measure the capillary network in the skin and retina, the sodium stores in the skin, and transepidermal water loss.
8. Serial studies on microcirculation in the skin and sodium content in the skin could help explain the pathogenesis of hypertension and potentially identify treatment approaches.

Microvascular rarefaction is a term that refers to the increase in peripheral resistance as a consequence of a decrease in the number of vessels in the microcirculation (arterioles and capillaries) per unit of tissue volume^5^. Direct intravital capillary video microscopy, which measures erythrocytes inside capillaries, provides a reliable and dynamic method to study skin capillaries. Several procedures have been used to study the nonperfused capillaries; for example, venous congestion increases the maximal capillary density and allows anatomic study of capillaries^6^. Studies with intravital video microscopy have demonstrated a 15% to 20% reduction in capillary density in nail-folds of patients with hypertension^7^. Prasad and colleagues used intravital fluorescein angiography and found a 20% reduction in capillary density in the forearm skin of hypertensive participants compared with normotensive participants^8^. This review will discuss the associations among microcirculation in the skin, sodium storage in the skin, and hypertension.

## Skin and Sodium

**Figure 1:**
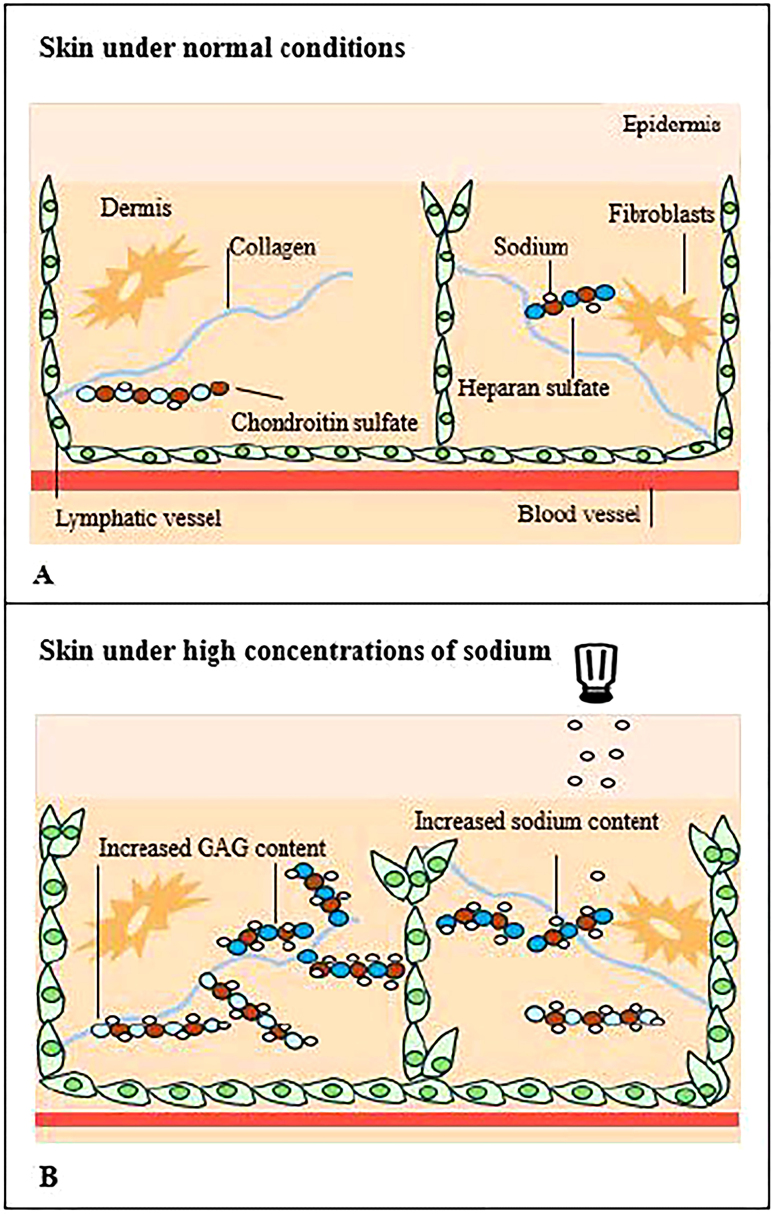
Schematic representation of sodium on the skin. Panel A shows normal conditions where GAGs such as chondroitin sulfate and heparan sulfate bind to sodium in the dermal interstitium. Panel B shows the increase in sodium content from sodium accumulation, an increase in the GAG content and synthesis as well as a decrease in blood vessel density. Additional changes include a decrease in VEGF-C levels, which limits lymphangiogenesis and possibly affects lymph vessel density. Created by JF.

The skin has two layers, the epidermis and the dermis. The dermis is an acellular layer containing fibroblasts, blood vessels, lymphatics, and nerves in an extracellular matrix of collagen, elastin, and GAGs (Figure 1). Most of the vessels are located in the papillary dermis 1 to 2 mm below the epidermal surface. The arterioles in the dermis have a diameter that ranges from 17 to 26 µm. GAGs in the dermis can bind sodium and provide non-osmotic storage of sodium in the interstitium due to highly negative charges^9^. Studies by Titze and colleagues suggested that in normal conditions, sodium binds to negatively charged GAGs in the dermal interstitium with no water retention. They found that skin sodium levels can increase up to 180–190 mmol/L without the expected increase in water content^10^. High concentrations of sodium increase GAG synthesis, extend the storage capacity of sodium, and facilitate the buffering system to avoid changes in systemic blood pressure^10^. But in conditions of high salt loading, the normal sodium-binding capacity of GAGs may be exceeded, and this causes interstitial hypertonicity. The skin interstitium’s role in sodium storage can contribute to blood pressure regulation^11^.

An increase in sodium load can cause several changes in this skin, and these include a decrease in blood vessel density^12,13^, an increase in sodium accumulation^10,14,15,16,17^, an increase in GAG content^10,15,16^, and increased inflammatory cell influx and lymph vessel density^16–18^. The skin interstitium may contribute to the pathogenesis of primary hypertension; this occurs when there are decreased levels of vascular endothelial growth factor-C (VEGF-C) in hypertensive patients, which limits lymphangiogenesis and reduces the protective function of the skin lymphatic system, which transfers electrolytes and water out of the skin^19^.

Chachaj and colleagues enrolled 131 patients who underwent skin biopsies before abdominal surgery^20^. The primary groups in this study were based on their arterial hypertension classification, and the secondary groups were based on sodium concentration in the skin. In both primary groups (i.e., increased blood pressure and normal blood pressure), there were no differences in sodium concentration in the skin, and this result differed from previous findings in which patients with hypertension had high sodium levels and lymphatic vessel density in the skin^19^. The authors suggested that patients with hypertension and high sodium levels have an interstitial buffering system in the skin with the following characteristics; it is saturated with sodium and water, has increased activation of transcription factor NFAT5 (nuclear factor of activated T cells 5) and VEGF-C pathways, and has increased lymphatic vessel density in the skin due to lymphangiogenesis. All of these events can occur in salt-sensitive hypertension.

Selvarajah et al. placed 48 healthy subjects on either a low sodium diet (70 mmol of sodium per day) or a high sodium diet (200 mmol of sodium per day) for 7 days^17^. Skin sodium and potassium concentrations were measured in biopsies and expressed as milligrams per gram of wet tissue. Sodium levels were 2.91 ± 0.08 mg/g tissue on the low sodium diet group and 3.12 ± 0.09 mg/g tissue on the high sodium diet group. Women but not men on the high sodium diet had a significant increase in mean blood pressure. There were no changes in mean VEGF-C levels in either men or women on the high sodium diet group compared to the low sodium diet group. However, there was a positive correlation between VEGF-C serum levels and the skin sodium to potassium ratio. These results suggest that the skin can buffer increased dietary sodium intake in men but not in women and that dietary sodium levels can influence vessel formation in the skin.

The following sections discuss rarefaction in the microcirculation in more detail.

## Changes in Microvascular Structure

The renin-angiotensin-aldosterone and the endothelin systems can cause endothelial dysfunction and vascular remodeling in vascular diseases and in aging. These events develop due to production of reactive oxygen species (ROS), inflammation, and cell growth and help explain the development of disorders in the vascular network^21^.

Both macro- and microcirculations have been studied in hypertension, and the term *cross-link* helps explain the effects of one circulation on the other^22^. Damage in vessels will vary based on their size; for example, small arteries will have functional defects, such as vasoconstriction and impaired vasodilatation, and structural defects like inward eutrophic remodeling. In large arteries, stiffening increases central systolic and pulse pressures, which are further enhanced by wave reflections due to structural adaptations in small resistance arteries^23^. Park and Schiffrin found that small artery remodeling is frequent and perhaps the earliest form of damage to target organs in patients with mild primary hypertension^24^.

Alterations in microcirculation may be initially related to increases in blood pressure during early morning hours, a period during sleep that is associated with an increased incidence of cardiovascular events^25^. Studies have demonstrated that in patients with primary hypertension, resistance arteries have increased media thickness and decreased lumen and external diameter resulting in an increased media-to-lumen ratio, but no change in total wall tissue, as shown by unchanged media cross-sectional area resulting in eutrophic remodeling. This results in a rearrangement of the same quantity of vessel wall components around a smaller vessel lumen without net cell growth^26^.

## Microvascular Rarefaction

Based on the work of several investigators, skin capillary rarefaction and reduction in the density of capillaries have been associated with hypertension^8,13,27^. Rarefaction is structural in origin and related to either impaired angiogenesis or capillary attrition. It is believed that these changes increase blood pressure by altering peripheral vascular resistance causing capillaries to gradually sustain damage and accumulate toxic reactive oxygen species^27,28^.

**Figure 2:**
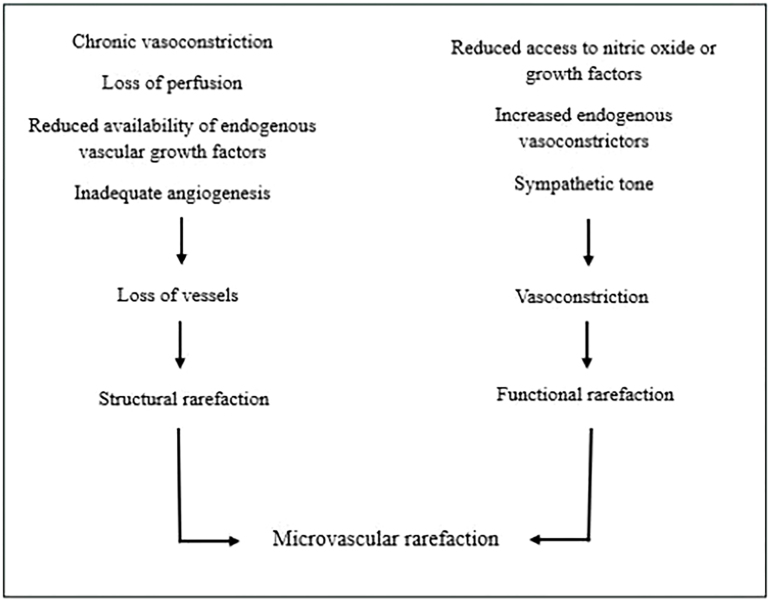
Representation of the factors involved in the development of microvascular rarefaction either due to factors related to structural rarefaction or those related to functional rarefaction.

Microvascular rarefaction can contribute to the increase in peripheral vascular resistance in hypertensive subjects, and several factors have been investigated^29^. Two different types of rarefaction have been found (Figure 2); *functional rarefaction* of microvessels is the result of vasoconstriction, secondary to reduced levels of nitric oxide (NO) or growth factors, such as growth hormone (GH), vascular endothelial growth factor (VEGF), and insulin-like growth factor 1 (IGF1), or increased levels of endogenous vasoconstrictors (e.g., endothelin and prostaglandins), or increased sympathetic tone. S*tructural rarefaction* is due to a loss of vessels in a vascular network and can occur due to chronic vasoconstriction and loss of perfusion, reduced levels of vascular growth factors, or inadequate angiogenesis. Structural rarefaction of capillaries and arterioles was detected in young spontaneously hypertensive rats before the elevation of blood pressure and in humans before the development of primary hypertension^27,28,29,30^.

Capillary rarefaction has also been studied with measurements of the retinal vasculature and retinal capillary flow. Patients with hypertension in early stages have lower retinal capillary density than normotensive subjects. Moreover, capillary rarefaction is increased in patients with chronic hypertension^31^. Therefore, widespread systemic microvascular rarefaction can be an important characteristic of early hypertension; in addition, current information suggests that capillary density can be an early factor in end-organ damage in hypertensive patients.

He et al. reported that in hypertensive patients, a modest reduction in salt intake improves dermal capillary density measured by capillaroscopy at the dorsa and sides of the fingers in a 12-week randomized double-blind crossover trial^13^. This occurred in different racial groups, including White, Black, and Asian patients, suggesting that salt intake is uniformly linked to microvascular rarefaction. The exact mechanisms on how salt affects the microcirculation remains uncertain, but experimental studies with rats suggest that salt intake increases hormonal vasoreactivity in the skin, possibly increasing the total peripheral resistance and causing blood pressure surges in those with salt-sensitive hypertension, which supports a possible relationship with osmotically inactive sodium accumulation^32^.

## Transepidermal Water Loss

The skin can transfer water to the environment by evaporation from its surface and through sweat glands. The outer layer of the epidermis controls the transcutaneous water loss, and the movement of water across the skin surface is controlled by flattened corneocytes that are surrounded by hydrophobic bilaminar lipids^33^, which include ceramides, cholesterol, and free fatty acids. This water loss can be measured with an open chamber device, an unventilated chamber device, or a condenser chamber device. Water loss depending on the skin area and is higher on the palms, soles, and axillae. It is increased with skin trauma and certain skin disorders such as atopic dermatitis.

Chen et al. measured sweat gland secretion and transepidermal water loss in patients with hypertension^34^. Skin biopsies were taken to determine sodium content. In addition, VEGF and hemoglobin in the skin were investigated as indices of skin micro circulation There was a positive correlation between sodium concentration in sweat and its concentration in the skin and between sodium concentration in sweat and age, but a negative correlation between sweat sodium and diastolic blood pressure. There was a positive correlation between the transepidermal water loss and sweat volume and between transepidermal water loss and surrogate measures for skin microvasculature based on VEGF-C levels and skin hemoglobin content. These results suggest that the skin participates in both fluid and sodium homeostasis, and changes in these functions potentially contribute to the development of hypertension.

Ogura et al. measured urinary concentration ability, body sodium/water balance, blood pressure, and transepidermal water loss in spontaneous hypertensive rats^35^. Spontaneous hypertension rats had significantly higher urine volume and lower urine osmolality. However, there was no significant difference between these rats and control rats in water intake, urinary electrolyte excretion, and plasma osmolarity. Spontaneously hypertensive rats had significantly higher blood pressure and skin sodium content but lower transepidermal water loss than control rats. Increased body temperature in spontaneous hypertensive rats increased total transepidermal water loss and significantly decreased blood pressure. These results suggest that decreased skin water loss can compensate for increased renal water loss. These changes are associated with vasoconstriction and hypertension. Consequently, understanding hypertension requires information about both renal function and skin function in terms of volume control and sodium storage.

Therefore, the skin can help regulate volume and potentially works in a reciprocal manner with the kidney^36^. For example, if urine output increases, the skin will decrease transepidermal water loss. Peripheral vasoconstriction decreases water loss through the skin but also increases blood pressure. Consequently, the skin contributes to normal physiology, potentially provides compensatory responses in patients with renal disease, and can contribute to the development and maintenance of hypertension. Sodium is stored in the interstitium of the skin, and this can be detected by ^23^Na-MRI magnetic resonance imaging. Sodium storage involves extracellular binding to GAG, and low storage levels in the skin does not result in an increase in tonicity. However, at high sodium levels there may be increased tonicity and increased fluid volumes in the skin. The exact details of skin sodium storage likely depend on sodium level in the skin, its thickness and lymphatic function. Understanding the contribution of transepidermal water loss to blood pressure control and volume control requires information about the regional distribution of transepidermal water loss, skin health, and factors that stimulate or inhibit water loss.

## Vascular Effects of Antihypertensive Drug Therapy

Antihypertensive drugs, such as dihydropyridine calcium channel blockers and renin-angiotensin system inhibitors (ACE inhibitors and angiotensin II receptor blockers), can prevent and decrease large artery stiffening and reverse microvascular remodeling^37,38^. These drugs improve microvascular structure and consequently reduce the media-to-lumen ratio; diuretics and beta-blockers do not alter the microvasculature^38,39,40^. Clinical studies have demonstrated that long term antihypertensive treatment increases capillary density in the skin of hypertensive patients without diabetes. The effects of angiotensin converting enzyme inhibition are mediated by activation of the bradykinin pathway, which stimulates VEGF formation and increases NO bioavailability^41^.

De Ciuceis and colleagues compared the effects of two drug combinations (lercanidipine **+** enalapril vs. lercanidipine **+** hydrochlorothiazide) on structural alterations in retinal arterioles, on skin capillary density, and on large artery distensibility in two small cohorts^42,43^. The capillary density increased only after treatment with lercanidipine **+** enalapril. The authors concluded that treatment with lercanidipine and enalapril for 24 weeks improves retinal arteriole structure and increases total capillary density and suggested that additional cohorts should be studied to validate their results. They believe that the hemodynamic and antioxidant effects of the medications could explain the positive effects on large arteries and on the structural alterations of retinal arterioles and capillaries. These drugs inhibit the mechanisms most likely involved in the intravascular stiffening and small artery remodeling, which include increased activity of the renin-angiotensin system, matrix metalloproteinases, intracellular signaling, and extracellular matrix formation^44^.

Zheleznyh et al. studied the effects of a fixed combination of an ACE inhibitor and diuretic on structural-functional parameters of the heart and vessels and on cognitive function. They used photoplethysmography and video capillaroscopy to evaluate the function and structure of capillaries in the fingers and found improved endothelial function of middle caliber arteries and microcirculatory vascular bed (p < 0.00005), increased density of skin capillary network at rest (p < 0.00007), and better cognitive function (p < 0.0001)^45^.

These studies and their methods provide an opportunity to understand the effect of antihypertensive medications on the microcirculation and raise the possibility that these changes in the microcirculation can function as a surrogate predictor of future cardiovascular events. Replication of these trials using larger study groups is needed to support the cross-link concept between the micro- and macrocirculations.

## Discussion

Ongoing research indicates that the skin functions as a third compartment of sodium distribution with a dynamic capacity for sodium storage and a buffering system that can vary based on salt consumption. The term *salt-sensitivity* has been used to identify subjects with changes of blood pressure with salt intake. The association between skin sodium content and blood pressure based on clinical and experimental studies is still controversial due to the mixed results^36^. Some studies report that skin sodium accumulation is associated with water retention and not with hypertonicity^46,47^, but other studies report that sodium accumulation is associated with hypertonicity and no water retention^3,4,18,48,49^.

Independent of factors associated with increased blood pressure, both functional and structural changes in systemic vasculature develop during established hypertension and involve both the micro- and macrovasculature. The supply of oxygen, nutrients, and metabolites occurs mostly in the microcirculation, which includes resistance arterioles, capillaries, and venules^50^. Changes such as an increased media-to-lumen ratio of small resistance arteries and increase vascular resistance are an initial adaptive response to hemodynamic load. The diameter and structure of small resistance arteries vary with fluctuations in blood pressure and flow patterns^30^; these variations are affected when microvascular rarefaction is present since this may affect not only peripheral vascular resistance but also muscle perfusion and metabolism^51^. It has been suggested that changes in the microvasculature can damage target organs secondary to increased hemodynamic load in hypertension^52,53^ and may be a predictor of future cardio-cerebrovascular events and complications secondary to hypertension^26^.

It is still uncertain how the accumulation of sodium in the skin can influence cutaneous blood flow and transepidermal water loss^36^. Jung and colleagues found that changes in the microcirculation occur first in nearly 93% of patients with a diagnosis of primary hypertension, long before organ dysfunctions become clinically apparent. Their findings show that the earliest disorders were discovered in skin capillaries and subsequently in the retina and the skeletal muscle^50^. An experimental study found a relationship among the precapillary skin arterioles of rats and high salt intake that explained the development of an increased hormonal vasoreactivity to angiotensin II. This up-regulation of hormonal sensitivity creates increased peripheral vascular resistance and thus increased afterload on the left ventricle^54^.

Recent studies demonstrate that patients benefit from a combination of antihypertensive medications, and have an improvement in their microcirculation, e.g., changes in the skin density measured by intravital microscopy. Stabilization of the small resistance artery structure occurs in hypertensive patients with long-term and effective therapy with either ACE inhibitors, angiotensin II receptor blockers, or calcium channel antagonists. Studies in patients with diabetes provide similar outcomes^55^. This promising information can help clinicians understand one of the most common clinical disorders worldwide and potentially determine which patients require close follow up and regular assessment of their treatments. Continued research with more participants is important to understand the pathogenesis and treatment of hypertension.

The skin also helps modulate body fluid levels through transepidermal water loss. This process depends on skin health, skin age, and the distribution of blood flow to the skin. There is limited information on the effect of antihypertensive medications on this physiological activity, and prospective studies in patients on these medications, especially in patients with chronic renal disease, could help explain responses to drug treatment in patients with hypertension.

## Conclusions

The structure of small arteries has been established as the best predictor of cardiovascular events in both primary and secondary hypertension. Recent studies led to a better understanding of the importance of the skin in blood pressure regulation. The development of skin capillary rarefaction has been studied in patients with primary hypertension, not only in the established phase, but also in early phases of hypertension when there are irregular elevations of blood pressure. This phenomenon can reduce the blood supply to the affected areas, leading to a decrease in the delivery of oxygen and nutrients and in the removal of metabolic waste. These changes denote an early phase in hypertension-mediated organ damage and could eventually cause angina, stroke, and renal dysfunction. Furthermore, microcirculation analysis provides a method for monitoring the beneficial effects of antihypertensive medications. Well-designed controlled studies with larger populations, including different ethnicities, are required since they could determine if different antihypertensive combinations have different effects on microcirculatory responses.

## Data Availability

All information in this review was collected from the references used for this review.
