# Supplementary material for: Skin microcirculation and hypertension: is there a connection?
Source: J Bras Nefrol. 2025 Jun 2;47(3):e202440192. doi: 10.1590/2175-8239-JBN-2024-0192en (PMC12129482; doi:10.1590/2175-8239-JBN-2024-0192en)
Supplement: Translation

# Translation: Microcirculação cutânea e hipertensão: existe uma relação?

**DOI:** 10.1590/2175-8239-JBN-2024-0192pt

**Authors:** Jackeline Flores, Camilo Pena, Kenneth Nugent

**Affiliations:** 1 Texas Tech University Health Sciences Center, School of Medicine, Department of Internal Medicine, Lubbock, Texas, United States.

**Keywords:** Sódio, Resistência Capilar, Hipertensão, Rarefação Microvascular

## Abstract

**Introdução:**

Introdução: Hipertensão é fator de risco importante para infarto do miocárdio, acidente vascular cerebral, doença arterial periférica e doença renal crônica. A OMS estimou que, globalmente, aproximadamente 1,28 bilhão de adultos apresentam hipertensão. Sua definição mudou ao longo dos anos e, em 2017, o *American College of Cardiology* e a *American Heart Association* definiram pressão arterial ≥ 130/80 mmHg como hipertensão.

**Métodos:**

Realizou-se pesquisa no banco de dados PubMed utilizando os descritores MeSH “sódio”, “resistência capilar”, “hipertensão”, “rarefação microvascular” para identificar artigos relevantes sobre níveis de sódio na pele, circulações periféricas e rarefação vascular, hipertensão.

**Resultados:**

Estudos experimentais em animais e humanos indicam que há menos capilares e arteríolas em pacientes com hipertensão primária, e a redução da densidade, também conhecida como rarefação, aumenta a resistência periférica. A densidade microvascular pode ser estimada de forma não invasiva por métodos como microscopia intravital por vídeo em regiões cutâneas ou pregas ungueais. Os mecanismos da redução da densidade foram estudados e podem ser usados como parâmetro na avaliação de opções terapêuticas. A pele fornece um reservatório de sódio e pode ajudar a moderar flutuações na pressão arterial associadas a mudanças na ingestão de sódio. Ademais, pode modular volumes de fluido no corpo pela perda de água transepidérmica.

**Conclusões:**

Esta revisão discute rarefação microvascular, alterações na estrutura da microvasculatura, incluindo capilares da pele com hipertensão, associação entre sódio e fisiologia da pele relacionada à hipertensão, perda de água transepidérmica e efeitos vasculares de alguns medicamentos anti-hipertensivos que contribuem para explicar seus benefícios na prevenção de eventos cardiovasculares.

## Introdução

Um em cada dois indivíduos com mais de 20 anos de idade nos Estados Unidos apresenta hipertensão e somente 39,6% dos pacientes hipertensos em uso de medicamentos têm a pressão arterial adequadamente controlada^1^. Diversos fatores contribuem para o desenvolvimento da hipertensão e estão associados à condição; entre eles estão o sistema nervoso autônomo, o sistema renina-angiotensina-aldosterona, o sistema da vasopressina e fatores ambientais, como ingestão de sal e estresse. Os eventos iniciais provavelmente envolvem a microcirculação; aumentos crônicos na pressão arterial desenvolvem-se secundariamente a alterações na microcirculação, incluindo vasoconstrição, hipóxia tecidual e redução da densidade arteriolar e capilar. Esses eventos elevam a resistência vascular periférica, o que pode causar lesão tecidual, iniciando-se com adaptações microvasculares que levam à lesão de órgãos^2^. A pele contém pequenas arteríolas e vênulas, além de sódio não osmótico, e pacientes com hipertensão apresentam níveis mais elevados de armazenamento de sódio na pele, mensurados por meio de ressonância magnética de sódio-23 (^23^Na-RM)^3,4^. Consequentemente, estudos sobre a microcirculação cutânea e seus níveis de sódio podem oferecer tanto *insights* quanto oportunidades para investigar a hipertensão. Os principais achados sobre esses mecanismos estão resumidos na Quadro 1.

**Quadro 1:** Principais afirmações sobre a microcirculação e a concentração de sódio na pele

1. A pele contém uma rede complexa de arteríolas, capilares e vênulas. Alterações anatômicas nas arteríolas e em sua densidade na pele, além de respostas fisiológicas a vasodilatadores e vasoconstritores, podem afetar a resistência vascular periférica e a pressão arterial.
2. Glicosaminoglicanos (GAGs) na derme ligam-se ao sódio e fornecem um local de armazenamento não osmótico de sódio no corpo.
3. Elevações dos níveis de sódio na pele podem reduzir a densidade vascular e aumentar o conteúdo de GAGs, o influxo de células inflamatórias e a densidade dos vasos linfáticos.
4. A rarefação microvascular pode ocorrer secundariamente a alterações funcionais em vasos com vasoconstrição e a modificações estruturais em vasos com remodelamento crônico. Essas alterações frequentemente manifestam-se na fase inicial da hipertensão, contribuindo para a elevação da pressão arterial.
5. A perda de água transepidérmica pode auxiliar na modulação dos volumes hídricos no corpo e atuar de forma recíproca com a função renal.
6. Os anti-hipertensivos - como os bloqueadores dos canais de cálcio do tipo diidropiridina, os inibidores da enzima conversora de angiotensina (ECA) e os bloqueadores dos receptores da angiotensina II - podem reduzir a rigidez das grandes artérias e reverter o remodelamento microvascular.
7. Os métodos experimentais contemporâneos permitem avaliar a rede capilar na pele e na retina, as reservas de sódio no tecido cutâneo e a perda de água transepidérmica.
8. Estudos em série sobre a microcirculação e a concentração de sódio na pele podem contribuir para elucidar a patogênese da hipertensão e, potencialmente, identificar abordagens terapêuticas.

A rarefação microvascular é um termo que designa o aumento da resistência periférica decorrente da redução do número de vasos na microcirculação (arteríolas e capilares) por unidade de volume tecidual^5^. A microscopia capilar intravital direta, que mensura os eritrócitos no interior dos capilares, constitui um método confiável e dinâmico para o estudo dos capilares cutâneos. Diversos procedimentos têm sido utilizados para avaliar capilares não perfundidos; por exemplo, a congestão venosa aumenta a densidade capilar máxima e permite o estudo anatômico dos capilares^6^. Estudos utilizando microscopia intravital demonstraram uma redução de 15% a 20% na densidade capilar nas pregas ungueais de pacientes hipertensos^7^. Prasad e colaboradores empregaram a angiografia fluoresceínica intravital e constataram uma redução de 20% na densidade capilar da pele do antebraço de participantes hipertensos em comparação com os normotensos^8^. Esta revisão abordará as associações entre a microcirculação cutânea, o armazenamento de sódio na pele e a hipertensão arterial.

## Pele e Sódio

A pele apresenta duas camadas: a epiderme e a derme. A derme é uma camada acelular que contém fibroblastos, vasos sanguíneos, vasos linfáticos e nervos, inseridos em uma matriz extracelular composta por colágeno, elastina e GAGs (Figura 1). A maioria dos vasos está localizada na derme papilar, a aproximadamente 1 a 2 mm abaixo da superfície epidérmica. As arteríolas na derme apresentam diâmetro que varia de 17 a 26 µm. Os GAGs na derme podem se ligar ao sódio, proporcionando armazenamento não osmótico deste íon no interstício devido às cargas altamente negativas^9^. Estudos realizados por Titze e colaboradores sugeriram que, em condições normais, o sódio liga-se a GAGs carregados negativamente no interstício dérmico sem retenção hídrica. Eles constataram que os níveis de sódio da pele podem aumentar até 180–190 mmol/L sem o aumento esperado no conteúdo de água^10^. Altas concentrações de sódio estimulam a síntese de GAG, ampliam a capacidade de armazenamento de sódio e facilitam o sistema tampão para evitar alterações na pressão arterial sistêmica^10^. Porém, em condições de elevada carga de sal, a capacidade normal de ligação de sódio dos GAGs pode ser excedida, ocasionando hipertonicidade intersticial. O papel do interstício da pele no armazenamento de sódio pode contribuir para a regulação da pressão arterial^11^.

**Figura 1:** Representação esquemática do sódio na pele. O painel A mostra condições normais, nas quais GAGs, como sulfato de condroitina e heparan sulfato, ligam-se ao sódio no interstício dérmico. O painel B ilustra o aumento no conteúdo de sódio decorrente do seu acúmulo, um aumento no conteúdo e na síntese de GAGs, bem como uma redução na densidade dos vasos sanguíneos. Outras alterações incluem a redução nos níveis de VEGF-C, que limita a linfangiogênese e possivelmente afeta a densidade dos vasos linfáticos. Elaborado por JF.

Um aumento na carga de sódio pode provocar diversas alterações nessa pele, incluindo uma redução na densidade dos vasos sanguíneos^12,13^, aumento no acúmulo de sódio^10,14–17^, aumento no conteúdo de GAG^10,15,16^, além de aumento no influxo de células inflamatórias e na densidade dos vasos linfáticos^16–18^. O interstício da pele pode contribuir para a patogênese da hipertensão primária; isso ocorre quando há níveis reduzidos do fator de crescimento endotelial vascular C (VEGF-C, do inglês *vascular endothelial growth factor-C*) em pacientes hipertensos, o que limita a linfangiogênese e compromete a função protetora do sistema linfático da pele - responsável pela remoção de eletrólitos e água do tecido cutâneo^19^.

Chachaj e colaboradores incluíram 131 pacientes submetidos a biópsias de pele antes de cirurgias abdominais^20^. Os grupos primários deste estudo foram definidos conforme a classificação da hipertensão arterial, enquanto os grupos secundários basearam-se na concentração de sódio na pele. Em ambos os grupos primários (ou seja, pressão arterial elevada e pressão arterial normal), não foram observadas diferenças nessa variável. Esse resultado difere de achados anteriores, nos quais pacientes hipertensos apresentaram níveis elevados de sódio e maior densidade de vasos linfáticos na pele^19^. Os autores sugeriram que pacientes com hipertensão e níveis elevados de sódio apresentam um sistema tampão intersticial na pele com as seguintes características: saturado com sódio e água, com aumento da ativação do fator de transcrição NFAT5 (fator nuclear de células T ativadas 5) e das vias de sinalização do VEGF-C, e com aumento da densidade de vasos linfáticos na pele devido à linfangiogênese. Todos esses eventos podem ocorrer na hipertensão sensível ao sal.

Selvarajah et al. submeteram 48 indivíduos saudáveis a uma dieta hipossódica (70 mmol de sódio por dia) ou a uma dieta hiperssódica (200 mmol de sódio por dia) durante 7 dias^17^. As concentrações de sódio e potássio na pele foram medidas em biópsias e expressas em miligramas por grama de tecido úmido. Os níveis de sódio foram de 2,91 ± 0,08 mg/g de tecido no grupo com dieta hipossódica e de 3,12 ± 0,09 mg/g de tecido no grupo da dieta rica em sódio. As mulheres, mas não os homens, submetidas à dieta rica em sódio apresentaram um aumento significativo na pressão arterial média. Não foram observadas alterações nos níveis médios de VEGF-C tanto em homens quanto em mulheres no grupo de dieta com alto teor de sódio em comparação ao grupo da dieta hipossódica. No entanto, houve uma correlação positiva entre os níveis séricos de VEGF-C e a proporção de sódio e potássio na pele. Esses resultados sugerem que a pele pode amortecer o aumento da ingestão dietética de sódio em homens, mas não em mulheres, e que os níveis de sódio da dieta podem influenciar a formação de vasos na pele.

As seções a seguir discutem mais detalhadamente a rarefação na microcirculação.

## Alterações na Estrutura Microvascular

Os sistemas renina-angiotensina-aldosterona e endotelina podem causar disfunção endotelial e remodelamento vascular em doenças vasculares e no envelhecimento. Esses eventos ocorrem devido à produção de espécies reativas de oxigênio (EROs), inflamação e proliferação celular, ajudando a explicar o desenvolvimento de distúrbios na rede vascular^21^.

Tanto a macro quanto a microcirculação têm sido estudadas na hipertensão, e o termo *cross-link* ajuda a explicar os efeitos de uma circulação sobre a outra^22^. Os danos aos vasos variam de acordo com seu tamanho; por exemplo, pequenas artérias apresentarão defeitos funcionais - como vasoconstrição e vasodilatação prejudicada - e defeitos estruturais - como remodelamento eutrófico para dentro. Nas grandes artérias, o enrijecimento eleva a pressão sistólica central e a pressão de pulso, que são ainda mais acentuadas por reflexões de onda decorrentes de adaptações estruturais em pequenas artérias de resistência^23^. Park e Schiffrin constataram que o remodelamento das pequenas artérias é frequente e, possivelmente, a forma mais precoce de lesão em órgãos-alvo em pacientes com hipertensão primária leve^24^.

Alterações na microcirculação podem estar inicialmente relacionadas a aumentos na pressão arterial durante as primeiras horas da manhã, um período durante o sono associado a uma maior incidência de eventos cardiovasculares^25^. Estudos demonstraram que, em pacientes com hipertensão primária, as artérias de resistência apresentam aumento da espessura da íntima-média e redução do lúmen e do diâmetro externo, resultando em um aumento da relação média-lúmen, mas não há alteração no tecido total da parede, conforme demonstrado pela área de secção transversal da íntima inalterada, resultando em remodelamento eutrófico. Isso resulta em um rearranjo da mesma quantidade de componentes da parede vascular ao redor de um lúmen vascular reduzido sem crescimento celular líquido^26^.

## Rarefação Microvascular

Com base no trabalho de diversos pesquisadores, a rarefação capilar da pele e a redução da densidade de capilares foram associadas à hipertensão^8,13,27^. A rarefação é de origem estrutural e está relacionada à angiogênese prejudicada ou ao desgaste capilar. Acredita-se que essas alterações elevem a pressão arterial por meio da modificação da resistência vascular periférica, levando os capilares a sofrerem danos graduais e acumularem espécies reativas de oxigênio tóxicas^27,28^.

A rarefação microvascular pode contribuir para o aumento da resistência vascular periférica em indivíduos hipertensos e diversos fatores foram investigados^29^. Dois tipos diferentes de rarefação foram encontrados (Figura 2); a *rarefação funcional* dos microvasos é o resultado da vasoconstrição, secundária a níveis reduzidos de óxido nítrico (NO) ou fatores de crescimento - como o hormônio do crescimento (GH, *growth hormone*), o fator de crescimento endotelial vascular (VEGF) e o fator de crescimento semelhante à insulina 1 (IGF1) - ou níveis aumentados de vasoconstritores endógenos (por exemplo, endotelina e prostaglandinas), ou aumento do tônus simpático. A *rarefação estrutural* se deve à perda de vasos em uma rede vascular e pode ocorrer devido à vasoconstrição crônica e perda de perfusão, níveis reduzidos de fatores de crescimento vascular ou angiogênese inadequada. A rarefação estrutural de capilares e arteríolas foi detectada em ratos jovens espontaneamente hipertensos antes da elevação da pressão arterial e em humanos antes do desenvolvimento de hipertensão primária^27–30^.

**Figura 2:** Representação dos fatores envolvidos no desenvolvimento da rarefação microvascular, seja por fatores relacionados à rarefação estrutural ou àqueles relacionados à rarefação funcional.

A rarefação capilar também foi estudada por meio de mensurações da vasculatura retiniana e do fluxo capilar na retina. Os pacientes com hipertensão em estágios iniciais apresentam densidade capilar retiniana reduzida em comparação com indivíduos normotensos. Além disso, a rarefação capilar é mais acentuada em pacientes com hipertensão crônica^31^. Portanto, a rarefação microvascular sistêmica generalizada pode ser uma característica importante da hipertensão precoce. Além disso, as informações atuais sugerem que a densidade capilar pode representar um fator precoce de lesão de órgãos terminais em pacientes hipertensos.

He et al. relataram que, em pacientes hipertensos, uma redução modesta na ingestão de sal melhorou a densidade capilar dérmica, medida por capilaroscopia na região dorsal e nas laterais dos dedos, em um ensaio clínico randomizado, duplo-cego e cruzado, com duração de 12 semanas^13^. Isso ocorreu em diferentes grupos raciais, incluindo pacientes brancos, pretos e asiáticos, sugerindo que a ingestão de sal está uniformemente ligada à rarefação microvascular. Os mecanismos exatos pelos quais o sal afeta a microcirculação permanecem incertos, mas estudos experimentais em ratos sugerem que a ingestão de sal aumenta a vasorreatividade hormonal na pele, possivelmente elevando a resistência vascular periférica total e causando picos pressóricos em pessoas com hipertensão sensível ao sal, o que sustenta uma possível relação com o acúmulo de sódio osmoticamente inativo^32^.

## Perda de Água Transepidérmica

A pele pode transferir água para o ambiente por meio da evaporação de sua superfície e através das glândulas sudoríparas. A camada mais externa da epiderme controla a perda de água transcutânea, e o movimento da água através da superfície da pele é regulado por corneócitos achatados que são cercados por lipídios bilaminares hidrofóbicos^33^, incluindo ceramidas, colesterol e ácidos graxos livres. Essa perda de água pode ser medida por meio de um dispositivo de câmara aberta, um dispositivo de câmara não ventilada ou um dispositivo de câmara condensadora. A perda hídrica depende da área da pele, sendo mais acentuada nas regiões palmares, plantares e axilares. Tal perda é aumentada em caso de trauma cutâneo e em determinadas doenças de pele, como a dermatite atópica.

Chen et al. avaliaram a secreção das glândulas sudoríparas e a perda de água transepidérmica em pacientes com hipertensão^34^. Foram realizadas biópsias de pele para determinar a concentração de sódio. Além disso, os níveis de VEGF e a hemoglobina no tecido cutâneo foram investigados como índices de microcirculação da pele. Observou-se uma correlação positiva entre a concentração de sódio no suor e sua concentração na pele, bem como entre a concentração de sódio no suor e a idade; porém, houve uma correlação negativa entre o sódio no suor e a pressão arterial diastólica. Houve uma correlação positiva entre a perda de água transepidérmica e o volume de suor e entre a perda de água transepidérmica e medidas substitutas para a microvasculatura cutânea, com base nos níveis de VEGF-C e na concentração de hemoglobina na pele. Esses resultados sugerem que a pele participa tanto da homeostase de fluidos quanto de sódio, e as alterações nessas funções podem contribuir para o desenvolvimento da hipertensão.

Ogura et al. mensuraram a capacidade de concentração urinária, o equilíbrio corporal de sódio/água, a pressão arterial e a perda de água transepidérmica em ratos espontaneamente hipertensos^35^. Ratos com hipertensão espontânea apresentaram volume urinário significativamente maior e menor osmolalidade da urina. No entanto, não houve diferença significativa entre esses animais e os ratos controle quanto à ingestão de água, excreção urinária de eletrólitos e osmolaridade plasmática. Ratos espontaneamente hipertensos apresentaram pressão arterial e concentração de sódio na pele significativamente mais elevados, porém menor perda de água transepidérmica em comparação aos ratos controle. O aumento da temperatura corporal nesses animais elevou a perda de água transepidérmica total e reduziu significativamente a pressão arterial. Esses resultados sugerem que a redução da perda de água pela pele pode compensar a perda aumentada de água pelos rins. Essas alterações estão associadas à vasoconstrição e à hipertensão. Consequentemente, a compreensão da hipertensão requer informações tanto sobre a função renal quanto sobre a função da pele em termos de controle de volume e armazenamento de sódio.

Portanto, a pele pode auxiliar na regulação do volume e, potencialmente, atuar de maneira recíproca com o rim^36^. Por exemplo, se o débito urinário aumentar, a pele reduzirá a perda de água transepidérmica. A vasoconstrição periférica diminui a perda de água pela pele, mas também eleva a pressão arterial. Consequentemente, a pele contribui para a fisiologia normal, potencialmente fornece respostas compensatórias em pacientes com doença renal e pode contribuir para o desenvolvimento e a manutenção da hipertensão. O sódio é armazenado no interstício da pele e pode ser detectado por ressonância magnética de sódio-23 (^23^Na-RM). O armazenamento de sódio envolve a ligação extracelular ao GAG, e baixos níveis de armazenamento na pele não resultam em aumento da tonicidade. Entretanto, em níveis elevados de sódio, pode haver aumento da tonicidade e dos volumes de fluidos na pele. Os detalhes exatos do armazenamento de sódio na pele provavelmente dependem do nível cutâneo de sódio, da espessura da pele e da função linfática. A compreensão da contribuição da perda de água transepidérmica para o controle da pressão arterial e do volume requer informações sobre a distribuição regional dessa perda, saúde da pele e fatores que estimulam ou inibem a perda de água.

## Efeitos Vasculares da Terapia com Medicamentos Anti-Hipertensivos

Os medicamentos anti-hipertensivos, como os bloqueadores dos canais de cálcio do tipo diidropiridina e os inibidores do sistema renina-angiotensina (inibidores da ECA e bloqueadores dos receptores da angiotensina II), podem prevenir e reduzir o enrijecimento das grandes artérias, além de reverter o remodelamento microvascular^37,38^. Esses medicamentos melhoram a estrutura microvascular e, consequentemente, reduzem a relação média/lúmen; diuréticos e betabloqueadores não alteram a microvasculatura^38–40^. Estudos clínicos demonstraram que o tratamento anti-hipertensivo de longo prazo aumenta a densidade capilar na pele de pacientes hipertensos sem diabetes. Os efeitos da inibição da enzima conversora da angiotensina são mediados pela ativação da via da bradicinina, a qual estimula a formação de VEGF e aumenta a biodisponibilidade de NO^41^.

De Ciuceis e colaboradores compararam os efeitos de duas combinações medicamentosas (lercanidipina + enalapril vs. lercanidipina + hidroclorotiazida) nas alterações estruturais das arteríolas da retina, na densidade capilar da pele e na distensibilidade das grandes artérias, em duas pequenas coortes^42,43^. O aumento da densidade capilar ocorreu somente após o tratamento com lercanidipina + enalapril. Os autores concluíram que o tratamento com lercanidipina e enalapril por 24 semanas melhora a estrutura das arteríolas da retina e aumenta a densidade capilar total, e sugeriram que coortes adicionais devem ser estudadas para validar seus resultados. Eles acreditam que os efeitos hemodinâmicos e antioxidantes dos medicamentos podem explicar os efeitos positivos sobre as grandes artérias e sobre as alterações estruturais das arteríolas e capilares da retina. Esses medicamentos inibem os mecanismos mais provavelmente envolvidos no enrijecimento intravascular e no remodelamento de pequenas artérias, que incluem o aumento da atividade do sistema renina-angiotensina, metaloproteinases da matriz, vias de sinalização intracelular e formação de matriz extracelular^44^.

Zheleznyh et al. estudaram os efeitos de uma combinação fixa de um inibidor da ECA e um diurético sobre parâmetros estruturais e funcionais do coração e dos vasos, bem como sobre a função cognitiva. Eles utilizaram fotopletismografia e videocapilaroscopia para avaliar a função e a estrutura dos capilares nos dedos e constataram uma melhora na função endotelial de artérias de médio calibre e no leito vascular microcirculatório (p < 0,00005), maior densidade da rede capilar da pele em repouso (p < 0,00007) e melhor função cognitiva (p < 0,0001)^45^.

Esses estudos e seus métodos oferecem uma oportunidade para compreender o efeito dos medicamentos anti-hipertensivos na microcirculação e levantam a possibilidade de que essas alterações na microcirculação possam funcionar como um preditor substituto de eventos cardiovasculares futuros. A replicação desses ensaios utilizando grupos de estudo maiores é necessária para apoiar o conceito de *cross-link* entre a micro e a macrocirculação.

## Discussão

Pesquisas em andamento indicam que a pele atua como um terceiro compartimento de distribuição de sódio, com uma capacidade dinâmica de armazenamento deste íon e um sistema tampão que pode variar de acordo com o consumo de sal. O termo *sensibilidade ao sal* tem sido utilizado para identificar indivíduos cuja pressão arterial apresenta alterações em função da ingestão de sal. A associação entre a concentração de sódio na pele e a pressão arterial, com base em estudos clínicos e experimentais, ainda é controversa devido aos resultados inconsistentes^36^. Alguns estudos relatam que o acúmulo de sódio na pele está associado à retenção de água e não à hipertonicidade^46,47^; porém, outros estudos relatam que esse acúmulo está associado à hipertonicidade e não à retenção de água^3,4,18,48,49^.

Independentemente dos fatores associados ao aumento da pressão arterial, mudanças funcionais e estruturais na vasculatura sistêmica se desenvolvem durante a hipertensão estabelecida, envolvendo tanto a micro quanto a macrovasculatura. O fornecimento de oxigênio, nutrientes e metabólitos ocorre predominantemente na microcirculação, que inclui arteríolas de resistência, capilares e vênulas^50^. Alterações como o aumento da relação média-lúmen de pequenas artérias de resistência e o aumento da resistência vascular constituem uma resposta adaptativa inicial à carga hemodinâmica. O diâmetro e a estrutura das pequenas artérias de resistência variam conforme as flutuações da pressão arterial e dos padrões de fluxo^30^. Essas variações são afetadas quando há rarefação microvascular, uma vez que esse fenômeno pode afetar não apenas a resistência vascular periférica, mas também a perfusão e o metabolismo musculares^51^. Estudos sugerem que alterações na microvasculatura podem causar lesões em órgãos-alvo de forma secundária ao aumento da carga hemodinâmica na hipertensão^52,53^, além de ser um preditor de futuros eventos cardiovasculares e complicações secundárias à hipertensão^26^.

Ainda não está claro como o acúmulo de sódio na pele pode influenciar o fluxo sanguíneo cutâneo e a perda de água transepidérmica^36^. Jung e colaboradores constataram que alterações na microcirculação ocorrem inicialmente em cerca de 93% dos pacientes com diagnóstico de hipertensão primária, muito antes de disfunções orgânicas se tornarem clinicamente aparentes. Seus achados demonstram que os primeiros distúrbios foram descobertos nos capilares da pele e, posteriormente, na retina e no músculo esquelético^50^. Um estudo experimental encontrou uma relação entre as arteríolas pré-capilares da pele de ratos e a ingestão elevada de sal, o que explicou o desenvolvimento de uma vasorreatividade hormonal aumentada à angiotensina II. Essa regulação positiva da sensibilidade hormonal gera aumento da resistência vascular periférica e, portanto, aumento da pós-carga no ventrículo esquerdo^54^.

Estudos recentes demonstram que os pacientes se beneficiam da combinação de medicamentos anti-hipertensivos e apresentam melhora na microcirculação como, por exemplo, alterações na densidade da pele avaliadas por microscopia intravital. A estabilização da estrutura das pequenas artérias de resistência ocorre em pacientes hipertensos submetidos a um tratamento eficaz e de longo prazo com inibidores da ECA, bloqueadores dos receptores da angiotensina II ou antagonistas dos canais de cálcio. Estudos em pacientes com diabetes apresentam desfechos semelhantes^55^. Essas informações promissoras podem auxiliar os médicos na compreensão de um dos distúrbios clínicos mais comuns em todo o mundo e, possivelmente, determinar quais pacientes demandam acompanhamento rigoroso e avaliação regular de seus tratamentos. A continuidade de pesquisas com um número maior de participantes é importante para a compreensão da patogênese e do tratamento da hipertensão.

A pele também contribui para a modulação dos níveis de fluidos corporais por meio da perda de água transepidérmica. Esse processo depende da saúde da pele, da idade cutânea e da distribuição do fluxo sanguíneo para a pele. As informações sobre o efeito dos medicamentos anti-hipertensivos sobre essa atividade fisiológica são limitadas e estudos prospectivos em pacientes em uso desses medicamentos - especialmente aqueles com doença renal crônica - podem ajudar a explicar as respostas ao tratamento medicamentoso em pacientes com hipertensão.

## Conclusões

A estrutura das pequenas artérias foi estabelecida como o melhor preditor de eventos cardiovasculares tanto na hipertensão primária quanto na secundária. Estudos recentes levaram a uma melhor compreensão da importância da pele na regulação da pressão arterial. O desenvolvimento da rarefação capilar cutânea foi estudado em pacientes com hipertensão primária, não apenas na fase estabelecida, mas também nas fases iniciais da hipertensão, quando há elevações irregulares da pressão arterial. Esse fenômeno pode reduzir o suprimento de sangue para as áreas afetadas, levando a uma redução na oferta de oxigênio e nutrientes e na remoção de resíduos metabólicos. Essas alterações denotam uma fase inicial de dano aos órgãos mediado pela hipertensão e podem, eventualmente, causar angina, acidente vascular cerebral e disfunção renal. Além disso, a análise da microcirculação fornece um método para monitorar os efeitos benéficos dos medicamentos anti-hipertensivos. São necessários estudos controlados bem desenhados com amostras populacionais maiores, incluindo diferentes etnias, uma vez que eles podem determinar se diferentes combinações de anti-hipertensivos exercem efeitos distintos sobre as respostas microcirculatórias.

## Data Availability

Todas as informações contidas nesta revisão foram coletadas a partir das referências bibliográficas utilizadas para esta revisão.
